# Safety of intracorporeal circular stapling esophagojejunostomy using trans-orally inserted anvil (OrVil™) following laparoscopic total or proximal gastrectomy - comparison with extracorporeal anastomosis

**DOI:** 10.1186/1477-7819-11-209

**Published:** 2013-08-23

**Authors:** Yoon Ju Jung, Dong Jin Kim, Jun Hyun Lee, Wook Kim

**Affiliations:** 1 Division of Gastrointestinal Surgery, Department of Surgery, Yeouido St. Mary’s Hospital, College of Medicine, The Catholic University of Korea, #62 Yeouido-dong, Yeongdeungpo-gu, Seoul 150-713, South Korea; 2 Division of Gastrointestinal Surgery, Department of Surgery, Bucheon St. Mary’s Hospital, College of Medicine, The Catholic University of Korea, Seoul, South Korea

**Keywords:** Laparoscopy, Total gastrectomy, Esophagojejunostomy, OrVil™

## Abstract

**Background:**

There have been several attempts to develop a unique and easier way to perform esophagojejunostomy during laparoscopy-assisted total gastrectomy or laparoscopy-assisted proximal gastrectomy. The OrVil™ system (Covidien, Mansfield, MA, USA) is one of those methods, but its technical and oncologic feasibility have not been proven and need to be observed.

**Methods:**

Among 87 patients who underwent laparoscopy-assisted total gastrectomy (LATG; 79 cases) and laparoscopy-assisted proximal gastrectomy with double tract anastomosis (LAPG_DT; 8 cases) from April 2004, 47 patients underwent the conventional extracorporeal method (Group I; 2004–2008) were compared with 40 patients treated with the intracorporeal OrVil™ system (Group II; 2009–2012).

**Results:**

There was no significant difference in clinicopathologic characteristics between the two groups except tumor location; more cardia lesions were involved in group II (p *=* 0.012). The mean time for esophagojejunostomy (E-J), defined as the time from anvil insertion to closure of the jejunal entry site has no significant difference (Group I vs II: 22.2 *±* 3.2 min vs 18.6 *±* 3.5 min, p *=* 0.623). In terms of anastomotic complication, there was no significant difference in E-J leakage and stricture. E-J leakage occurred in 2 out of 47 (4.3%) cases in group I and 2 out of 40 (5%) in group II (p = 0.628); half of them were treated conservatively in each group and the others underwent reoperation. E-J stricture occurred in 2 (4.3%) cases in group I and 1 (2.5%) in group II (p = 0.561), which required postoperative gastrofiberscopic balloon dilatation.

**Conclusions:**

Esophagojejunostomy using the OrVil™ system was a feasible and safe technique compared with the conventional extracorporeal method through mini-laparotomy in terms of anastomotic complications. Moreover, it can help to reduce surgeon’s stress regarding esophagojejunostomy because it needs no purse-string procedure and serves a secure operation view laparoscopically.

## Background

Laparoscopy-assisted total gastrectomy (LATG) has not become popular because of difficulties in lymph node dissection around the distal splenic vessels (no. 11d) and splenic hilum (no. 10) and complex reconstruction procedures. Of these, reconstruction after LATG has been regarded as the most stressful problem for surgeons. During LATG, most surgeons prefer conventional esophagojejunostomy (E-J) through a mini-laparotomy at the upper epigastrium, which is similar to open reconstruction [1,2]. In addition, laparoscopy-assisted proximal gastrectomy with double-tract anastomosis (LAPG-DT) also needs E-J [3]. A relatively large wound is necessary to perform secure anastomotic processes consisting of preparing the jejunal limb, making a purse-string suture, inserting the anvil head and fastening the anvil to the abdominal esophagus. Above all, insertion of the anvil into the esophagus through a mini-laparotomy is a troublesome and time-consuming procedure, which may lead to postoperative anastomosis leakage, intra-abdominal abscess and subsequent sepsis especially in the patients with high body mass index or short abdominal esophagus. For these reasons, various types of E-J reconstruction methods have been introduced [4-7]. However, these methods require specialized instruments or experienced surgical skills, and there is still no standard method for E-J reconstruction after LATG or LAPG-DT. The OrVil™ system (Covidien, Mansfield, MA, USA) was recently introduced for E-J reconstruction during LATG. It is a new anastomotic instrument that omits the intracorporeal insertion of the anvil and purse-string sutures. We evaluated its surgical safety and feasibility for E-J reconstruction with a comparison to conventional extracorporeal reconstruction during LATG and LAPG-DT.

## Methods

### Patients and methods

This study was approved by institutional review board of Yeouido St. Mary’s hospital as the subject number, XC12RIMI0062S. Among 87 patients who underwent LATG or LAPG-DT from April 2004 to December 2012 in a single hospital, 47 patients underwent the conventional method (group I; 2004 to 2008) and were compared with 40 patients treated with the OrVil™ system (group II; 2009 to 2012). The clinicopathologic findings and surgical outcomes related to the E-J procedure were analyzed.

### Operative procedures for LATG

**Figure 1:**
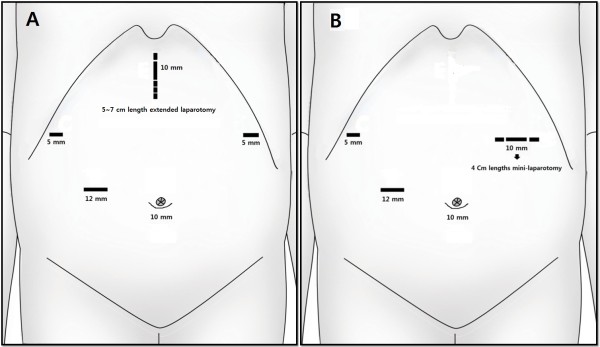
**Placement of the trocars in each procedure. (A)** Extracorporeal reconstruction. **(B)** Intracorporeal reconstruction using the OrVil™ system.

Typically, one periumbilical trocar (10 mm) was inserted using Hasson’s method, and four additional trocars were introduced under laparoscopic guidance according to Figure 1. A flexible laparoscope was then introduced through the umbilical port. With a pneumoperitoneum of 12 to 14 mmHg, the left lobe of the liver was retracted by suturing the *pars flaccida*. The greater omentum was divided with ultrasonic shears at the mid-portion of the transverse colon at 4 to 5 cm away from the gastroepiploic arcades toward the lower pole of the spleen in cases of early gastric cancer (EGC). The roots of the left gastroepiploic vessels were then exposed and divided with clips. Several short gastric vessels were dissected with ultrasonic shears, and the greater curvature side was completely mobilized from the splenic hilum and left side of the diaphragm. The right gastroepiploic vein and artery were divided individually at their roots with clips, and clearing of the pancreatic head area was done. The right gastric artery was then exposed and divided at its origin with clips after dissection of the anterior portion of the hepatoduodenal ligament. The duodenum was transected at 2 cm distal from the pylorus with a linear stapler. Lymph node-bearing soft tissues along the common hepatic artery, proximal splenic artery and celiac axis were dissected. The root of the left gastric artery was exposed and divided with double clips. Meticulous dissection was continued along the splenic vessels to clear the No. 11p and 11d lymph nodes. In cases of EGC, No. 10 lymph node dissection was not performed. The perigastric lymph nodes were dissected along the lesser curvature up to the esophagogastric junction with division of both *vagus nervi*. The abdominal esophagus was then freely mobilized after dissection of the No.1 and 2 lymph nodes. Then the following procedures were followed depending on the group.

### Operative procedures for LAPG-DT

**Figure 2:**
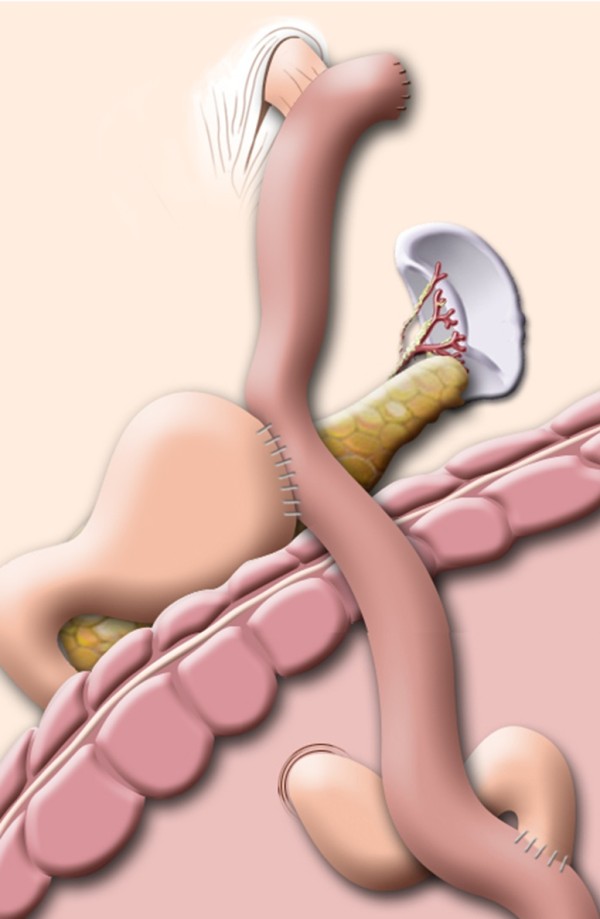
Illustration of the final reconstruction after laparoscopy-assisted proximal gastrectomy with double-tract anastomosis.

We have performed LAPG-DT since December 2011 for EGC located at the upper third of the stomach. Most of the procedures for mobilization and dissection of the stomach were similar to LATG except for preservation of the right gastroepiploic and right gastric vessels. Following full mobilization of the abdominal esophagus, esophageal transaction was done with a laparoscopic linear stapler and an anvil was inserted. Esophagojejunostomies in all LAPG-DT procedures were performed intracorporeally using OrVil™. Gastrojejunostomy was made at the Roux-limb 15 to 20 cm distal from the E-J and the jejunojejunostomy was made at a point 20 cm distal from the gastrojejunostomy. Gastrojejunostomy and jejunojejunostomy were performed using the hand-sewing method through the left upper quadrant (LUQ) trocar extension wound. The final reconstruction of the LAPG-DT is illustrated in Figure 2.

### Conventional extracorporeal esophagojejunostomy

In cases of extracorporeal anastomosis, an approximately 5 to 7 cm upper midline mini-laparotomy was made, and the stomach was then pulled through the wound (Figure 1A). Esophageal transection was performed using the purse-string clamp (US Surgical Corporation, Norwalk, CT, USA), and the anvil of a circular stapler (21 or 25 mm) (US Surgical Corporation) was inserted into the esophageal stump. A Roux limb of the jejunum was prepared and anastomosed to the esophageal stump in an end-to-side manner. The entry site of the jejunal stump was closed using a linear stapler, and a jejunojejunostomy was performed extracorporeally.

### Intracorporeal esophagojejunostomy using the OrVil™ system

**Figure 3:**
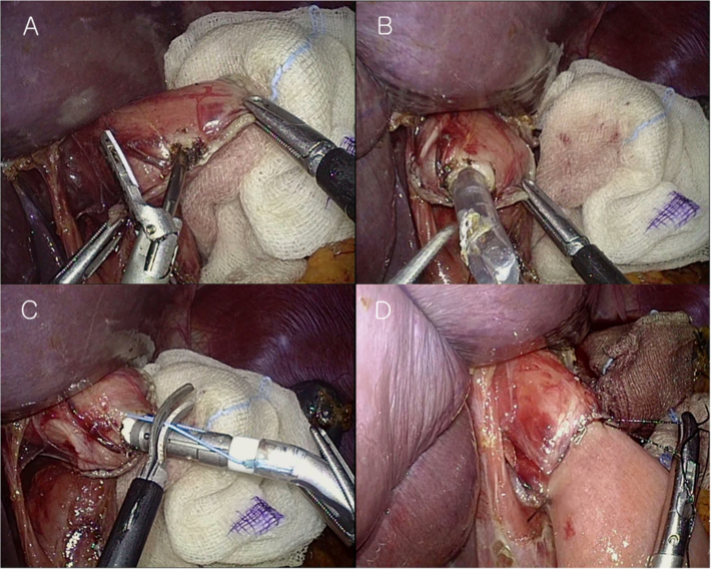
**Procedures of anvil insertion through the OrVil**^**™ **^**system. (A)** Making the entry hole at the transected esophageal stump with the ultrasonic shear device. **(B)** The orogastric tube was introduced into the abdominal cavity. **(C)** Cutting the thread connecting the anvil shaft and the orogastric tube. **(D)** Making the reinforcement suture intracorporeally, including the double stapling point.

In cases of intracorporeal anastomosis, esophageal transection was performed with an endoscopic linear stapler (US Surgical Corporation), and the OrVil™ tube was then transorally introduced into the esophagus by the anesthesiologist. OrVil™ was introduced through the patient’s mouth with the rubbery orogastric tube at first. As the operator identified the OrVil™ tube reaching the esophageal stump, a small hole was made on the esophageal stump (Figure 3A). The tube was then extracted through the hole until the anvil reached the esophageal stump (Figure 3B). When the pre-tilted anvil is introduced into the mouth, the anesthesiologist should protect and confirm that the anvil is inserted through the upper esophageal sphincter under the laryngoscopic view. Then, the tube was disconnected from the anvil by cutting the connecting thread and removed from the abdominal cavity (Figure 3C). The 10-mm trocar site at the LUQ was extended to about 4 cm in length (Figure 1B), and through it the circular stapler was introduced. The shaft of the circular stapler was introduced into the jejunum, and E-J was performed in the same way as the extracorporeal Roux-en-Y E-J using the intracorporeal approach. Reinforcement sutures were made, including the double stapling point (Figure 3D).

## Results

### Clinicopathologic characteristics

The clinicopathologic findings were not significantly different between the two groups, with the exception of the tumor location (*P =* 0.012). More cardia lesions were observed in group II (Table 1).

**Table 1:** Clinicopathologic characteristics according to anastomosis type

		**Group I**	**Group II**	***P-***
		**(n = 47)**	**(n = 40)**	**value**
Age^a^		61.2 *±* 12.1	63.4 *±* 12.1	0.399
Gender (male:female)		37:10	31:9	0.891
Body mass index (kg/m^2^)		23.4 *±* 4.3	24.0 *±* 1 4.8	0.452
Tumor location	E-G Junction	2 (4.3)	0 (0)	0.012
	Cardia	21 (44.7)	32 (80)	
	High body	19 (40.4)	6 (15)	
	Whole	5 (10.7)	2 (5)	
Stage (UICC 7th)	Ia	14 (28.9)	18 (45)	0.404
	Ib	5 (10.6)	7 (17.5)	
	IIa	7 (14.9)	4 (10)	
	IIb	4 (8.5)	2 (5)	
	IIIa	5 (10.6)	3 (7.5)	
	IIIb	12 (25.5)	5 (12.5)	
	IIIc	0 (0)	1 (2.5)	
Depth of invasion	T1a	3 (6.4)	12 (30)	0.060
	T1b	11 (23.4)	8 (20)	
	T2	8 (17)	6 (15)	
	T3	13 (27.7)	6 (15)	
	T4a	12 (25.5)	8 (20)	
Lymph node metastasis	0	27 (57.4)	29 (72.5)	0.290
	1	2 (4.3)	3 (7.5)	
	2	5 (10.6)	4 (10)	
	3a	4 (8.5)	2 (5)	
	3b	9 (19.1)	2 (5)	
Tumor size (cm)^a^		5.3 *±* 3.6	4.5 *±* 3.2	0.323
Retrieved nodes (number)^a^		36.6 *±* 17.8	41.1 *±* 18.4	0.117
Metastatic nodes (number)^a^		6.6 *±* 11.4	2.3 *±* 6.1	0.029
Proximal resected margin (cm)^a^		2.8 *±* 1.1	2.5 *±* 1.7	0.328
Extent of dissection	D1+	1 (2.1)	18 (45)	<0.001
	D2	47 (97.9)	22 (55)	
Combined resection	Spleen	3 (6.5)	5 (12.5)	0.277
	Gallbladder	2 (4.3)	0 (0)	

^a^Continuous variables are expressed as the mean *±* SD. Other variables are expressed as number of cases (percentage). *E-G*, esophagogastric junction; *UICC*, Union for International Cancer Control.

### Surgical outcomes

The total operation time (group I versus group II: 261.5 *±* 77.3 minutes versus 220.2 *±* 65.2 minutes, *P =* 0.067) and the mean time for the E-J (defined as the time from anvil insertion to end-to-side E-J to closure of the jejunal entry site) (group I versus group II: 22.2 *±* 3.2 minutes versus 18.6 *±* 3.5 minutes, *P =* 0.623) in group II was slightly shorter than that of group I but not statistically significant. Postoperative anastomosis-related complications occurred in four out of forty-seven patients (8.6%) in group I and three out of forty patients (7.5%) in group II, with no significant difference between groups (*P =* 0.863). Additionally, there was no significant difference in E-J leakage, which occurred in two out of forty-seven (4.3%) patients in group I and two out of forty (5%) in group II (*P =* 0.628); half of them were treated conservatively in each group. E-J stricture occurred in two (4.3%) patients in group I and one (2.5%) in group II (*P =* 0.921), which required postoperative gastrofiberscopic balloon dilatation. Liquid oral intake, first flatus and postoperative hospital stay were not significantly different between the groups (Table 2).

**Table 2:** Surgical outcomes and complications related to the anastomosis procedures

	**Group I (n = 47)**	**Group II (n = 40)**	***P-*value**
Operation time (minutes)^a^	261.5 *±* 77.3	220.2 *±* 65.2	0.067
Time for anastomosis (minutes)^a^	22.2 *±* 3.2	18.6 *±* 3.5	0.623
Anastomosis-related complications^b^	4 (8.6)	3 (7.5)	0.863
E-J leakage	2 (4.3)	2 (5)	0.628
E-J stricture	2 (4.3)	1 (2.5)	0.561
Time to first flatus (days)^a^	3.3 *±* 0.8	3.2 *±* 0.6	0.391
First diet (days)^a^	3.7 *±* 1.6	3.1 *±* 0.6	0.530
Postoperative hospital stay (days)^a^	12.3 *±* 5.6	11.6 *±* 2.3	0.588

^a^Continuous variables are expressed as the mean *±* SD.

^b^Nominal variables are expressed as number of cases (percentage). *E-J*, esophagojejunostomy.

## Discussion

Gastric cancer is one of the most common types of malignant tumor in Korea, and laparoscopic-assisted distal gastrectomy (LADG) has been well-established as an alternative modality for the treatment of EGC patients. However, LATG has not become as popular as LADG because of the low incidence of upper gastric cancer and the technical difficulties of the laparoscopic approach, particularly for safe E-J anastomosis and complete lymph node dissection around the splenic vessel and hilum. Neither has LAPG been performed because of the high incidence of post-operative morbidity associated with the anastomosis, such as reflux esophagitis and anastomosis stricture. However, recent studies have revealed novel anastomosis techniques to overcome those complications. Double-tract anastomosis is one of them, requiring extracorporeal or intracorporeal E-J. An E-J can be performed after LATG by means of either extracorporeal or intracorporeal methods. Of these two methods, extracorporeal reconstruction has been typically preferred by most surgeons because no specific device is required and it is an optimal procedure for reconstruction following LATG.

The typical extracorporeal E-J anastomosis is similar to conventional open surgery, including the application of the anvil to the abdominal esophagus after purse-string suturing by hand-sewing or using a purse-string device. However, these procedures are challenging in contrast to open surgery because the narrow operating window might not be large enough for anvil insertion and purse-string suturing, particularly in patients who are obese or have a large left lobe of the liver. In this situation, a longer laparotomy is required, which might weaken the merits of the laparoscopic approach. For this reason, several modified reconstructive methods by means of a mini-laparotomy have been introduced [5-7]. A side-to-side E-J anastomosis has been introduced through a mini-laparotomy using an endoscopic linear stapler instead of a circular stapler [7]. However, this procedure might not result in adequate proximal margins when the tumor location is higher, and it carries the risk of anastomotic stenosis. Conversely, some procedures for purse-string suturing to the abdominal esophagus were introduced with the use of the circular stapler. Asao *et al.*[1] introduced a newly developed purse-string suture device (Takasago Medical, Tokyo, Japan), and Takiguchi *et al.*[8] introduced a simple laparoscopic purse-string suture technique using a semiautomatic suturing device (Endostich; Covidien, Mansfield, MA, USA). Although simple and secure reconstruction techniques were reported to facilitate the use of intra-corporeal E-J for LATG, there were no comparisons with either procedure. For this reason, many surgeons still prefer extracorporeal E-J anastomosis by means of mini-laparotomy.

In our series, extracorporeal anastomosis was replaced with an intracorporeal procedure from 2009 to overcome such difficulties during extracorporeal procedures. We typically performed intracorporeal reconstruction using the OrVil™ system. It is a commercially available device consisting of a 21-mm or 25-mm anvil with the head pre-tilted and the tip attached to an 18-Fr orogastric tube. While several other intracorporeal reconstruction procedures using the circular stapler have required more complex procedures, including purse-string suturing and anvil insertion into the abdominal esophagus [9,10], it was inserted trans-orally and did not require purse-string suturing, which is the most stressful procedure for the surgeon. Theoretically, it appears to be a non-stressful procedure, but it could also be time-consuming and challenging if the surgeon is unfamiliar with it. Additionally, there is a risk of abdominal esophageal injury during the anvil insertion, and the security of the purse-string suture is not guaranteed. For this reason, many surgeons still prefer extracorporeal E-J with a mini-laparotomy.

In terms of early surgical outcomes, anastomotic leakage occurred in two patients in each group, which was not significantly different; one of these from each group required reoperation and the other two patients recovered with conservative management. The OrVil™ system has been shown be safe compared to extracorporeal or intracorporeal reconstructive methods and can be a feasible modality [6,9].

Contrary to what was expected, intracorporeal reconstruction using the OrVil™ system was not faster than the extracorporeal procedure in this study. Although the time for reconstruction gradually shortened with experience, it appears that other procedures, namely, preparing for the Roux limb, insertion of the circular stapler and docking of the Roux limb to the OrVil™ system, might influence the operation time. However, it is true that this procedure reduced the surgeon’s stress regarding the laparoscopy and intracorporeal purse-string sutures, particularly for surgeons who are less experienced. Jeong *et al*. reported 16 cases of successful intracorporeal E-J anastomosis using a circular stapler and a trans-orally inserted anvil: the OrVil™ [11]. In this study, the trans-oral placement of the anvil simplified the procedure and reduced the operative time. Although the study size was small and the follow up was relatively short, there were no perioperative complications (for example, leakage or stenosis) associated with the anastomosis.

There might be some concerns about using the OrVil™ system. Although this method theoretically permits an esophagojejunal stapled anastomosis with a 25-mm circular stapler, surgeons could encounter some difficulties when they introduce the OrVil™ through the narrowed site of the lower esophagus, such as in the larynx or at the tracheal bifurcation level. When the OrVil tube is introduced, if there is any resistance, careful traction and inspection of the location of the anvil with respect to the larynx are required. There might be the risk of esophageal injury during anvil insertion, and disconnection of the anvil from the tube, particularly in the upper esophageal sphincter area if the operator overlooks the potential problem, especially for patients who have a small body, short neck or small chin. Actually, although we had no experience of esophageal injury, there was one case of anvil disconnection from the tube at the upper esophageal sphincter area and it was removed by endoscopy. Applying OrVil™ in such patients, we should manipulate the orogastric tube gently with caution. In addition, if there is resistance on pulling out the OrVil™ tube, this may be caused by a physiologically narrow space. In such cases, we found that waiting for a minute helped overcome the resistance and secure the placement of the anvil. The other potential problem in this study was infection following the introduction of a contaminated instrument through the oral cavity into the abdominal cavity. To prevent an abdominal infection, we immediately removed the tube without contact with the peritoneum, and performed copious saline irrigation. Postoperative abdominal infection did not occur.

## Conclusions

The OrVil™ system was relatively feasible and safe with esophagojejunostomy in LATG or LAPG-DT. Moreover, it was more useful and convenient in the patients in whom anvil insertion may be difficult.

## Consent

Our study is retrospective study, and Institutional review board of Catholic medical college approved our study including omit additional informed consent. However all of our patients agreed using their medical information.

## Abbreviations

EGC: Early gastric cancer; E-J: Esophagojejunostomy; LADG: Laparoscopic-assisted distal gastrectomy; LAPG-DT: Laparoscopy-assisted proximal gastrectomy with double-tract anastomosis; LATG: Laparoscopy-assisted total gastrectomy; LUQ: Left upper quadrant.

## Competing interests

The authors declare that they no competing interests.

## Authors’ contributions

KW carried out surgery and organized all of this study. KDJ and LJH participated in the study design and revised the manuscript. JYJ drafted and wrote this manuscript. All authors read and approved the final manuscript.
